# Continuous Glucose Monitoring‐Derived Glycemic Phenotypes Reflect Metabolic Heterogeneity Linked to Advanced Cardiovascular‐Kidney‐Metabolic Syndrome in Older Adults With Type 2 Diabetes

**DOI:** 10.1111/1753-0407.70298

**Published:** 2026-11-10

**Authors:** Hang Su, Jiaying Ni, Jinghao Cai, Jingyi Guo, Yufei Wang, Xiaojing Ma, Jingyi Lu, Jian Zhou, Feng Jiang

**Affiliations:** ^1^ Department of Endocrinology and Metabolism Shanghai Sixth People's Hospital Affiliated to Shanghai Jiao Tong University School of Medicine, Shanghai Clinical Center for Diabetes, Shanghai Diabetes Institute, Shanghai Key Laboratory of Diabetes Mellitus Shanghai China; ^2^ Clinical Research Center, Shanghai Sixth People's Hospital Affiliated to Shanghai Jiao Tong University School of Medicine Shanghai China

**Keywords:** cardiovascular‐kidney‐metabolic syndrome, continuous glucose monitoring, glycemic phenotype, older adults with diabetes

## Abstract

**Background:**

To investigate the metabolic characteristics of continuous glucose monitoring (CGM)‐derived glycemic phenotypes and their association with cardiovascular‐kidney‐metabolic (CKM) syndrome in older adults with Type 2 diabetes.

**Methods:**

Clinical and biochemical data, including estimated β‐cell function and insulin resistance, along with CGM data, were collected from 2491 adults aged ≥ 60 years with Type 2 diabetes. CKM syndrome was staged according to the American Heart Association criteria, with Stages 3–4 defined as advanced CKM syndrome. CGM‐derived glycemic phenotypes were constructed using an outcome‐guided workflow combining XGBoost feature‐importance analysis with correlation analysis and clinical interpretability.

**Results:**

The prevalence of advanced CKM syndrome was 65.0% in our population. Using an outcome‐guided and clinically interpretable workflow, time above range (TAR_>10.0_) and mean amplitude of glycemic excursions (MAGE) were selected to represent hyperglycemic burden and glycemic variability, respectively, and were used to construct two‐dimensional CGM‐derived glycemic phenotypes. Four phenotypes were constructed through this framework and differed significantly in glycemic and metabolic profiles. Although the high TAR_>10.0_/high MAGE phenotype showed the greatest hyperglycemic burden and glycemic variability (*p* < 0.001), the high TAR_>10.0_/low MAGE phenotype showed the largest point estimate of association with advanced CKM syndrome among the four phenotypes (OR 1.62, 95% CI 1.16–2.27). Notably, this phenotype also exhibited the lowest insulin sensitivity among the four phenotypes.

**Conclusions:**

CGM‐derived glycemic phenotypes demonstrated distinct metabolic profiles and differential associations with advanced CKM syndrome, suggesting that CGM data may help inform personalized management strategies for older adults with Type 2 diabetes.

## Highlights

- Four continuous glucose monitoring (CGM)‐derived phenotypes integrating hyperglycemic burden and glycemic variability exhibited distinct metabolic profiles in older adults with Type 2 diabetes.
- The phenotype characterized by high hyperglycemic burden and low variability showed the lowest insulin sensitivity and the largest point estimate of association with advanced cardiovascular‐kidney‐metabolic syndrome, despite not having the highest HbA1c.
- CGM‐derived glycemic patterns may provide additional information for characterizing metabolic heterogeneity beyond conventional glycemic indicators.

## Graphical abstract

In 2491 older adults with Type 2 diabetes, four continuous glucose monitoring‐derived glycemic phenotypes reflected metabolic heterogeneity. Despite not having the highest HbA1c, the phenotype with high hyperglycemic burden and low glycemic variability showed greater insulin resistance and the largest point estimate of association with advanced cardiovascular‐kidney‐metabolic syndrome after HbA1c adjustment (OR 1.62, 95% CI 1.16–2.27).

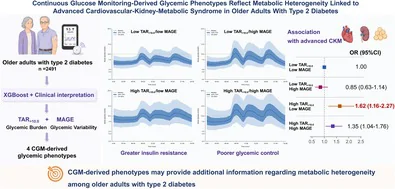

**Abbreviations**
2hPG: 2‐h postprandial plasma glucose
ADRR: average daily risk range
BMI: body mass index
CGM: continuous glucose monitoring
CI: confidence interval
CKD‐EPI: Chronic Kidney Disease Epidemiology Collaboration
CKM: cardiovascular‐kidney‐metabolic
CONGA: continuous overall net glycemic action
CV: coefficient of variation
eGFR: estimated glomerular filtration rate
FPG: fasting plasma glucose
GLP‐1: glucagon‐like peptide‐1
HbA1c: glycated hemoglobin A1c
HDL: high‐density lipoprotein cholesterol
HOMA2‐B: homeostasis model assessment 2 of β‐cell function
HOMA2‐IR: homeostasis model assessment 2 of insulin resistance
HOMA2‐S: homeostasis model assessment 2 of insulin sensitivity
IQR: interquartile range
LAGE: largest amplitude of glycemic excursions
LDL: low‐density lipoprotein cholesterol
MAG: mean absolute glucose change
MAGE: mean amplitude of glycemic excursions
MODD: mean of daily differences
MSG: mean sensor glucose
OR: odds ratio
PREVENT: Predicting Risk of Cardiovascular Disease EVENTs
SBP: systolic blood pressure
SD: standard deviation
T2DM: Type 2 diabetes mellitus
TAR: time above range
TBR: time below range
TG: triglycerides
TIR: time in range
TITR: time in tight range
TyG: triglyceride‐glucose index
UACR: urinary albumin‐to‐creatinine ratio

## 1 Introduction

Cardiovascular‐kidney‐metabolic (CKM) syndrome provides an integrated framework for understanding the interconnected pathways linking metabolic, kidney, and cardiovascular disorders across a continuum of disease progression [1, 2]. This framework recognizes excess and dysfunctional adipose tissue as an early metabolic abnormality, with insulin resistance, dysglycemia, kidney dysfunction, and cardiovascular disease occurring as related features along the CKM continuum. Type 2 diabetes represents an important manifestation of metabolic dysfunction within this continuum and is closely linked to increased risks of cardiovascular and kidney disease [1]. In individuals with Type 2 diabetes, abnormalities in glucose regulation occur within a broader metabolic context characterized by heterogeneous cardiometabolic profiles [1, 2]. Although glycated hemoglobin A1c (HbA1c) is widely used for assessing long‐term glycemic exposure, it provides limited information on dynamic glucose patterns and interindividual heterogeneity in glucose regulation [3, 4, 5].

In recent years, continuous glucose monitoring (CGM) studies have shown that individuals with similar HbA1c levels may still exhibit distinct glucose patterns [4, 5], thereby expanding the concept of glycemic phenotypes in diabetes [6, 7]. Recent studies, including our previous work, have used CGM data to identify glycemic phenotypes associated with different metabolic features, such as muscle or hepatic insulin resistance, β‐cell dysfunction, and impaired incretin action [8, 9]. Insulin resistance is also a recognized component of CKM pathophysiology [1, 2]. These findings suggest that CGM‐derived glycemic patterns may reflect interindividual heterogeneity in glucose regulation and be associated with underlying metabolic characteristics. However, many existing phenotype classifications are based on complex algorithms, which may limit their clinical interpretability. A simpler and more interpretable approach is therefore needed to retain key glycemic phenotype information while facilitating clinical applicability. Whether CGM‐derived glycemic phenotypes are associated with metabolic characteristics and advanced CKM syndrome (Stages 3–4) among individuals with Type 2 diabetes remains unclear.

Exploring metabolic heterogeneity across glycemic phenotypes is particularly relevant in older adults with Type 2 diabetes. With advancing age, the burden of CKM syndrome increases substantially [10, 11]. At the same time, age‐related β‐cell dysfunction, reduced peripheral insulin sensitivity, and sarcopenia may contribute to more complex patterns of glucose exposure and variability [12, 13]. However, direct evidence on CGM‐derived glycemic phenotypes in this population is still limited.

Therefore, in this study of older adults with Type 2 diabetes, we characterized different CGM‐derived glycemic phenotypes, evaluated their metabolic characteristics, and examined associations with advanced CKM syndrome.

## 2 Research Design and Methods

### 2.1 Study Participants

This study included older adults with Type 2 diabetes who underwent standardized assessment of diabetic complications and glucose monitoring at Shanghai Sixth People's Hospital Affiliated to Shanghai Jiao Tong University School of Medicine [14]. Participants were eligible if they were aged 60 years or older and had maintained a stable treatment regimen during the preceding 3 months. A stable treatment regimen was defined as no changes in the classes or prescribed doses of glucose‐lowering medications during this period. Patients were excluded if they met any of the following criteria: (1) acute diabetic complications before or during hospitalization, such as diabetic ketoacidosis; (2) glucocorticoid therapy; (3) malignancy or psychiatric disorders; and (4) severe hepatic dysfunction (defined as alanine aminotransferase and/or aspartate aminotransferase levels ≥ 3 times the upper limit of normal) or dialysis. Finally, 2491 participants enrolled between May 2020 and July 2023 had complete clinical data and were eligible for CKM syndrome classification. The study was approved by the Ethics Committee of Shanghai Sixth People's Hospital Affiliated to Shanghai Jiao Tong University School of Medicine and was conducted in accordance with the Declaration of Helsinki (approval No. 2020‐KY‐024[K]). All participants provided written informed consent prior to inclusion in the study.

### 2.2 Clinical Covariates

Medical history and medication use were obtained from electronic medical records. Diabetic complications and cardiovascular conditions were assessed based on clinical evaluations performed during hospitalization and relevant information from electronic medical records. All participants underwent comprehensive physical examinations, including measurements of height, weight, and blood pressure. On the second day of hospitalization, venous blood samples were collected after a 10‐h overnight fast to measure HbA1c, fasting plasma glucose (FPG), fasting insulin, fasting C‐peptide, lipid profiles, serum creatinine, and other biochemical parameters. Two‐hour postprandial glucose (2hPG), insulin, and C‐peptide were measured 2 h after breakfast under the standardized dietary protocol. All laboratory measurements were performed using standard methods [14]. Albuminuria was assessed using the urinary albumin‐to‐creatinine ratio (UACR).

Homeostasis model assessment 2 (HOMA2) indices, including HOMA2‐B, HOMA2‐IR, and HOMA2‐S, were calculated from FPG and C‐peptide concentrations using the HOMA calculator (University of Oxford, Oxford, UK) to estimate β‐cell function, insulin resistance, and insulin sensitivity, respectively [15, 16]. C‐peptide was used instead of insulin to better reflect endogenous insulin secretion in participants receiving insulin therapy. FPG and C‐peptide values outside the accepted input ranges of the HOMA2 calculator were capped at the corresponding upper or lower limits [17].

Type 2 diabetes was diagnosed according to the 2010 American Diabetes Association Standards of Medical Care in Diabetes [18]. Body mass index (BMI) was calculated as weight divided by height squared (kg/m^2^). Estimated glomerular filtration rate (eGFR) was calculated using the Chronic Kidney Disease Epidemiology Collaboration (CKD‐EPI) equation [19].

### 2.3 CGM Measurements

All participants underwent blinded retrospective CGM monitoring using the iPro 2 system (Medtronic) from the day of admission for 7 days to record subcutaneous interstitial glucose levels. The CGM system recorded 288 glucose values per day, with measurements obtained every 5 min. Finger‐stick capillary glucose values were measured with a SureStep glucose meter (LifeScan) and entered for calibration at least every 12 h. The mean CGM data completeness was 99.75%. During CGM monitoring, participants continued their original treatment regimens and followed a standardized diet providing 25 kcal/kg/day, with carbohydrates, protein, and fat accounting for 55%, 17%, and 28% of total energy, respectively. Breakfast, lunch, and dinner provided 20%, 40%, and 40% of daily energy intake and were scheduled at 06:30–07:30, 11:00–12:00, and 17:00–18:00, respectively [14].

CGM‐derived metrics reflecting glucose levels, glucose variability, and hypoglycemia exposure were calculated [20]. These included time in range (TIR), time in tight range (TITR), time above range (TAR_>7.8_, TAR_>10.0_, and TAR_>13.9_), time below range (TBR_<3.9_ and TBR_<3.0_), mean sensor glucose (MSG), standard deviation (SD), coefficient of variation (CV), mean amplitude of glycemic excursions (MAGE), interquartile range (IQR), largest amplitude of glycemic excursions (LAGE), mean absolute glucose change (MAG), mean of daily differences (MODD), continuous overall net glycemic action calculated at 1‐h and 4‐h intervals (CONGA1h and CONGA4h), average daily risk range (ADRR), and J‐index. The definitions and calculation methods of these metrics are shown in Supplementary Table 1.

### 2.4 Definition of CKM Syndrome

CKM stage was determined according to the 2023 American Heart Association framework [1]. Because Type 2 diabetes fulfills a criterion for Stage 2, all participants in this study were classified within Stages 2–4. Among participants without established cardiovascular disease, Stage 3 was assigned in the presence of either very‐high‐risk chronic kidney disease, defined according to eGFR and albuminuria, or a PREVENT‐estimated 10‐year cardiovascular disease risk of ≥ 20% (Supplementary Table 2). Stage 4 was defined by documented coronary heart disease, stroke, or peripheral artery disease. Participants who met neither the Stage 3 nor Stage 4 criteria remained classified as Stage 2. Advanced CKM syndrome was defined as Stages 3–4 [11].

### 2.5 Statistical Analysis

Statistical analyses were performed using R 4.2.2 and Python 3.10. The normality of continuous variables was assessed. Non‐normally distributed continuous variables were presented as medians (interquartile ranges) and compared using Wilcoxon rank‐sum or Kruskal‐Wallis tests. Categorical variables were expressed as number (%) and compared using the *χ* ^2^ test. Trends across ordered groups were evaluated using the Jonckheere‐Terpstra test for continuous variables and the Cochran‐Armitage trend test for categorical variables.

A machine learning‐assisted workflow combining outcome‐guided selection with clinical interpretability was used to identify representative CGM metrics for glycemic phenotype construction. Multiple CGM metrics were included as candidate features, with advanced CKM syndrome as the outcome. An XGBoost model was applied with five‐fold cross‐validation, and gain‐based feature importance was used to rank the relative contribution of candidate CGM metrics [21]. Spearman correlation analysis was performed to assess correlations among glycemic metrics and to identify highly correlated or overlapping indicators. Representative CGM metrics were selected by jointly considering feature importance, inter‐metric correlations, and clinical interpretability. Based on the selected metrics, glycemic phenotypes were subsequently constructed.

The resulting phenotypes were compared with respect to fasting and postprandial metabolic measurements and other clinical characteristics. Univariable and multivariable logistic regression models were used to evaluate the associations between glycemic phenotypes and advanced CKM syndrome. Odds ratios (ORs) and 95% confidence intervals (CIs) were calculated. Three models were constructed sequentially: Model 1 was the unadjusted model; Model 2 was adjusted for age, sex, diabetes duration, BMI, and current smoking status; and Model 3 was further adjusted for HbA1c. Additional models were constructed with further adjustment for HOMA2‐IR or glucose‐lowering treatment. All *p* values were two‐sided, and *p* < 0.05 was considered statistically significant.

## 3 Results

### 3.1 Study Participant Characteristics

Among the 2491 older adults with Type 2 diabetes included in this study, 872, 721, and 898 were classified as CKM Stages 2, 3, and 4, respectively (Table 1). The median age was 67.0 (64.0–72.0) years, and the median HbA1c was 8.1% (7.0%–9.6%). Participants in higher CKM stages tended to be older, have longer diabetes duration, higher systolic and diastolic blood pressure, triglyceride levels, and HbA1c, and lower low‐density lipoprotein cholesterol and eGFR (all *p* for trend < 0.05). The proportions of participants receiving antihypertensive, lipid‐lowering, and antiplatelet therapies also differed significantly across CKM stages (all *p* overall < 0.001). Among CGM metrics, MSG and TAR_>10.0_ increased across CKM stages (both *p* for trend < 0.01), while TIR and TITR decreased progressively (both *p* for trend < 0.001).

**TABLE 1:** Characteristics of participants.

	Total	CKM Stage 2	CKM Stage 3	CKM Stage 4	*p* overall	*p* trend
*N*	2491	872	721	898		
Sex, *n* (%)					< 0.001	< 0.001
Male	1329 (53.4%)	351 (40.3%)	478 (66.3%)	500 (55.7%)		
Female	1162 (46.6%)	521 (59.7%)	243 (33.7%)	398 (44.3%)		
Age, years	67.0 (64.0, 72.0)	64.0 (62.0, 67.0)	70.0 (67.0, 74.0)	68.0 (65.0, 73.0)	< 0.001	< 0.001
Diabetes duration, years	15.0 (9.0, 21.0)	14.0 (8.0, 20.0)	15.0 (9.0, 20.0)	17.0 (10.0, 23.0)	< 0.001	< 0.001
Systolic blood pressure, mmHg	135.0 (125.0, 147.0)	130.0 (120.0, 140.0)	140.0 (130.0, 152.0)	137.0 (126.0, 150.0)	< 0.001	< 0.001
Diastolic blood pressure, mmHg	79.0 (72.0, 85.0)	78.0 (71.0, 82.0)	80.0 (74.0, 87.0)	78.0 (71.0, 85.0)	< 0.001	0.012
Body mass index, kg/m^2^	24.1 (22.0, 26.1)	23.5 (21.6, 25.6)	24.4 (22.6, 26.7)	24.2 (22.2, 26.3)	< 0.001	< 0.001
eGFR, mL/min/1.73 m^2^	87.9 (74.0, 94.7)	92.8 (83.3, 96.8)	82.7 (67.0, 90.9)	85.6 (69.9, 93.3)	< 0.001	< 0.001
Total cholesterol, mmol/L	4.3 (3.6, 5.2)	4.5 (3.8, 5.3)	4.6 (3.8, 5.3)	3.9 (3.2, 4.8)	< 0.001	< 0.001
Triglycerides, mmol/L	1.2 (0.9, 1.7)	1.1 (0.8, 1.6)	1.3 (0.9, 1.9)	1.2 (0.9, 1.7)	< 0.001	0.003
HDL cholesterol, mmol/L	1.1 (0.9, 1.3)	1.2 (1.0, 1.4)	1.0 (0.9, 1.2)	1.1 (0.9, 1.3)	< 0.001	< 0.001
LDL cholesterol, mmol/L	2.4 (1.8, 3.1)	2.5 (2.0, 3.2)	2.6 (2.1, 3.3)	2.1 (1.6, 2.8)	< 0.001	< 0.001
HbA1c, %	8.1 (7.0, 9.6)	7.8 (6.8, 9.2)	8.3 (7.2, 9.7)	8.3 (7.2, 9.7)	< 0.001	< 0.001
FPG, mmol/L	6.5 (5.4, 7.9)	6.4 (5.4, 7.7)	6.6 (5.5, 8.0)	6.6 (5.4, 8.0)	0.129	0.116
2hPG, mmol/L	10.9 (8.5, 13.8)	10.6 (8.1, 13.4)	10.8 (8.6, 13.7)	11.4 (8.9, 14.6)	< 0.001	< 0.001
Current smokers, *n* (%)	414 (16.6%)	81 (9.3%)	181 (25.1%)	152 (16.9%)	< 0.001	< 0.001
Oral glucose‐lowering drugs, *n* (%)	1454 (58.4%)	532 (61.0%)	413 (57.3%)	509 (56.7%)	0.142	0.066
Metformin, *n* (%)	873 (35.0%)	324 (37.2%)	266 (36.9%)	283 (31.5%)	0.021	0.013
Sulfonylureas, *n* (%)	224 (9.0%)	91 (10.4%)	59 (8.2%)	74 (8.2%)	0.181	0.108
Thiazolidinediones, *n* (%)	49 (2.0%)	18 (2.1%)	14 (1.9%)	17 (1.9%)	0.965	0.796
Glinides, *n* (%)	82 (3.3%)	35 (4.0%)	26 (3.6%)	21 (2.3%)	0.122	0.048
DPP‐4 inhibitors, *n* (%)	367 (14.7%)	141 (16.2%)	115 (16.0%)	111 (12.4%)	0.043	0.023
α‐Glucosidase inhibitors, *n* (%)	699 (28.1%)	261 (29.9%)	187 (25.9%)	251 (28.0%)	0.209	0.360
SGLT2 inhibitors, *n* (%)	413 (16.6%)	137 (15.7%)	110 (15.3%)	166 (18.5%)	0.154	0.115
Insulin, *n* (%)	1775 (71.3%)	544 (62.4%)	526 (73.0%)	705 (78.5%)	< 0.001	< 0.001
GLP‐1 receptor agonists, *n* (%)	72 (2.9%)	22 (2.5%)	19 (2.6%)	31 (3.5%)	0.450	0.242
Antihypertensive agents, *n* (%)	1372 (55.1%)	270 (31.0%)	506 (70.2%)	596 (66.4%)	< 0.001	< 0.001
Lipid‐lowering agents, *n* (%)	1878 (75.4%)	630 (72.2%)	462 (64.1%)	786 (87.5%)	< 0.001	< 0.001
Antiplatelet agents, *n* (%)	854 (34.3%)	132 (15.1%)	120 (16.6%)	602 (67.0%)	< 0.001	< 0.001
Mean sensor glucose, mmol/L	8.7 (7.8, 9.9)	8.4 (7.6, 9.5)	8.8 (7.9, 9.9)	9.1 (8.0, 10.2)	< 0.001	< 0.001
Standard deviation, mmol/L	2.4 (1.9, 3.0)	2.3 (1.8, 2.9)	2.4 (1.9, 3.0)	2.5 (2.0, 3.1)	< 0.001	< 0.001
Coefficient of variation, %	27.1 (22.7, 32.1)	26.8 (22.5, 31.9)	27.0 (22.4, 31.8)	27.4 (23.1, 32.4)	0.192	0.085
MAGE, mmol/L	5.3 (4.1, 6.7)	5.2 (4.1, 6.5)	5.5 (4.1, 6.7)	5.5 (4.3, 6.8)	0.026	0.007
TAR_>13.9_, %	3.0 (0.0, 10.0)	2.0 (0.0, 8.0)	3.0 (0.0, 10.0)	5.0 (0.0, 13.0)	< 0.001	< 0.001
TAR_>10.0_, %	27.0 (13.0, 42.0)	21.0 (10.0, 37.0)	28.0 (15.0, 43.0)	31.0 (16.0, 47.0)	< 0.001	< 0.001
TAR_>7.8_, %	57.0 (42.0, 75.0)	53.0 (37.0, 69.0)	60.0 (43.0, 77.0)	62.0 (45.0, 79.0)	< 0.001	< 0.001
TIR, %	72.0 (57.0, 86.0)	78.0 (62.0, 88.0)	71.0 (57.0, 84.0)	68.0 (53.0, 83.0)	< 0.001	< 0.001
TITR, %	42.0 (24.0, 57.0)	47.0 (30.0, 62.0)	40.0 (22.0, 55.0)	38.0 (21.0, 54.0)	< 0.001	< 0.001
TBR_<3.9_, %	0.0 (0.0, 1.0)	0.0 (0.0, 1.0)	0.0 (0.0, 1.0)	0.0 (0.0, 1.0)	0.338	0.148
TBR_<3.0_, %	0.0 (0.0, 0.0)	0.0 (0.0, 0.0)	0.0 (0.0, 0.0)	0.0 (0.0, 0.0)	0.048	0.014

*Note:* Data are presented as median (interquartile range) or number (percentage) unless otherwise indicated.

Abbreviations: 2hPG, 2‐h postprandial plasma glucose; CKM, cardiovascular‐kidney‐metabolic; DPP‐4, dipeptidyl peptidase‐4; eGFR, estimated glomerular filtration rate; FPG, fasting plasma glucose; GLP‐1, glucagon‐like peptide‐1; HbA1c, glycated hemoglobin A1c; HDL, high‐density lipoprotein; LDL, low‐density lipoprotein; MAGE, mean amplitude of glycemic excursions; SD, standard deviation; SGLT2, sodium‐glucose cotransporter 2; TAR _>7.8_, time above range > 7.8 mmol/L (>140 mg/dL); TAR_>10.0_, time above range > 10.0 mmol/L (>180 mg/dL); TAR_>13.9_, time above range > 13.9 mmol/L (>250 mg/dL); TBR _<3.0_, time below range < 3.0 mmol/L (< 54 mg/dL); TBR _<3.9_, time below range < 3.9 mmol/L (< 70 mg/dL); TITR, time in tight range; TIR, time in range.

### 3.2 Feature Importance and Correlations of CGM Metrics

XGBoost feature‐importance analysis showed that TAR_>10.0_, a marker of hyperglycemic burden, had the highest model importance among CGM metrics for distinguishing advanced CKM syndrome (Figure 1A). Based on these results and considerations of clinical interpretability, we then performed correlation analyses (Figure 1B). First, HbA1c showed moderate correlations with CGM metrics reflecting glycemic exposure, with inverse correlations for TIR and TITR (*r* = −0.56 and −0.50, respectively) and positive correlations for TAR_>10.0_ and MSG (*r* = 0.57 for both; all *p* < 0.001).

**FIGURE 1:**
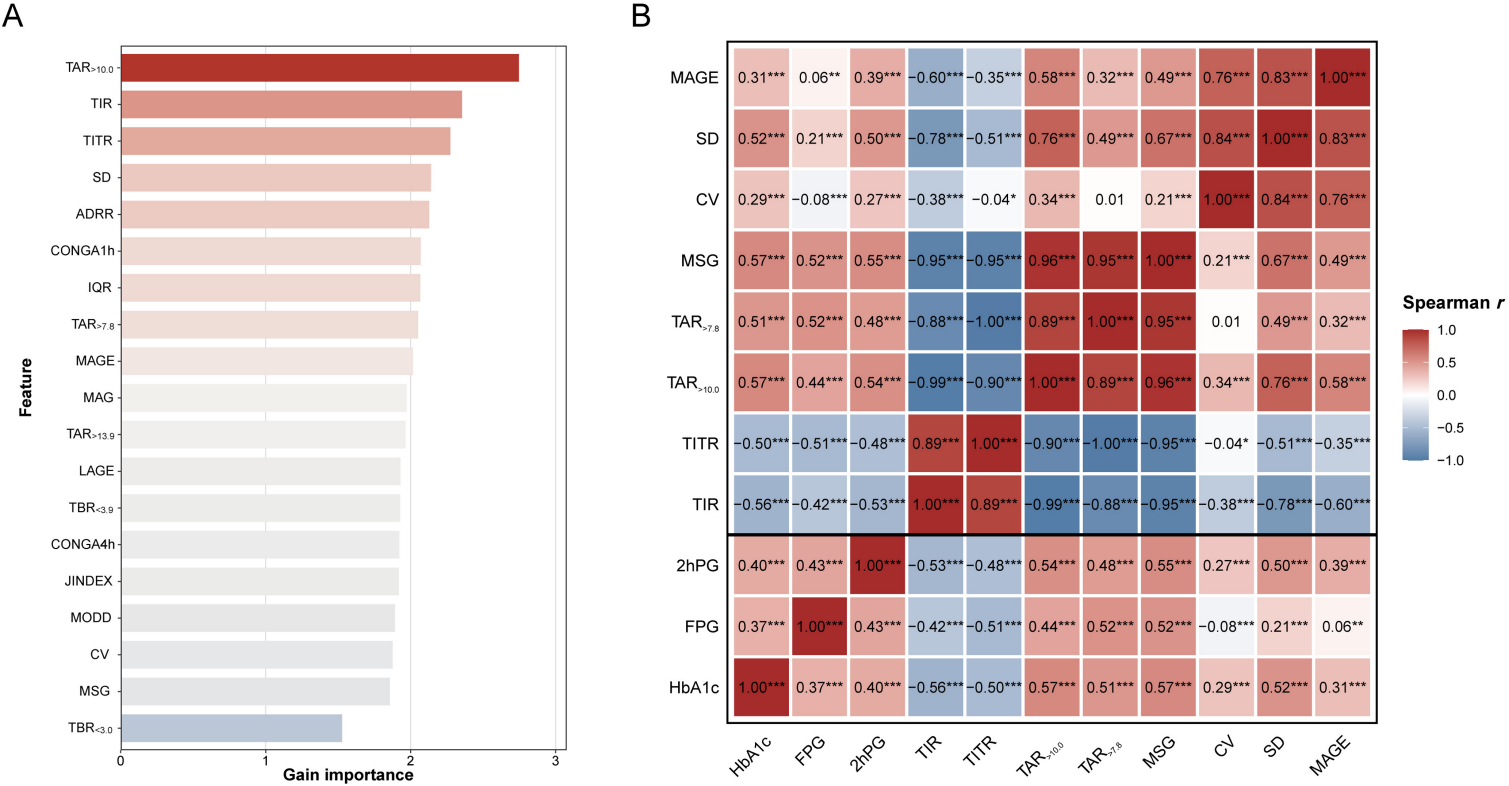
Machine learning‐based feature selection and correlation structure of glycemic metrics. (A) XGBoost‐based gain importance of candidate CGM‐derived metrics for advanced CKM syndrome. (B) Spearman correlation matrix showing the correlation structure among traditional glycemic indicators and CGM‐derived metrics. Colors indicate the direction and magnitude of correlation coefficients. *p < 0.05; **p < 0.01; ***p < 0.001. Abbreviations: 2hPG, 2‐h postprandial plasma glucose; ADRR, average daily risk range; CGM, continuous glucose monitoring; CKM, cardiovascular‐kidney‐metabolic; CONGA, continuous overall net glycemic action; CV, coefficient of variation; FPG, fasting plasma glucose; HbA1c, glycated hemoglobin A1c; IQR, interquartile range; LAGE, largest amplitude of glycemic excursions; MAG, mean absolute glucose change; MAGE, mean amplitude of glycemic excursions; MODD, mean of daily differences; MSG, mean sensor glucose; SD, standard deviation; TAR, time above range; TBR, time below range; TIR, time in range; TITR, time in tight range.

Within CGM metrics, indicators from the same domain showed strong correlations. In the glycemic burden domain, TAR_>10.0_ was highly correlated with TIR, MSG, and TITR (*r* = −0.99, 0.96, and −0.90, respectively; all *p* < 0.001). Similarly, variability metrics, including CV, SD, and MAGE, were strongly and positively correlated with each other, with correlation coefficients of approximately 0.80 (all *p* < 0.001).

We further assessed correlations between CGM metrics from different domains. TAR_>10.0_ was strongly correlated with SD, the top‐ranked variability metric in the XGBoost model (*r* = 0.76, *p* < 0.001), suggesting substantial information overlap. Among other clinically interpretable metrics in the glycemic variability domain, MAGE ranked higher than CV in the XGBoost analysis and showed a more moderate correlation with TAR_>10.0_ (*r* = 0.58, *p* < 0.001). Therefore, considering its feature importance, clinical interpretability, and lower overlap with TAR_>10.0_ than SD, MAGE was selected to represent glycemic variability and was combined with TAR_>10.0_ to construct the glycemic phenotypes.

### 3.3 Glycemic and Metabolic Features Across Glycemic Phenotypes

Participants were then classified into four CGM‐derived glycemic phenotypes according to the median cutoffs of TAR_>10.0_ (27.0%) and MAGE (5.3 mmol/L, 95 mg/dL). The corresponding CGM profiles are shown in Figure 2. Metabolic characteristics differed substantially across these phenotypes (Supplementary Table 3). The low TAR_>10.0_/low MAGE phenotype showed the most favorable overall glycemic control, with the lowest HbA1c and the highest TIR (Figure 3; both *p* < 0.001).

**FIGURE 2:**
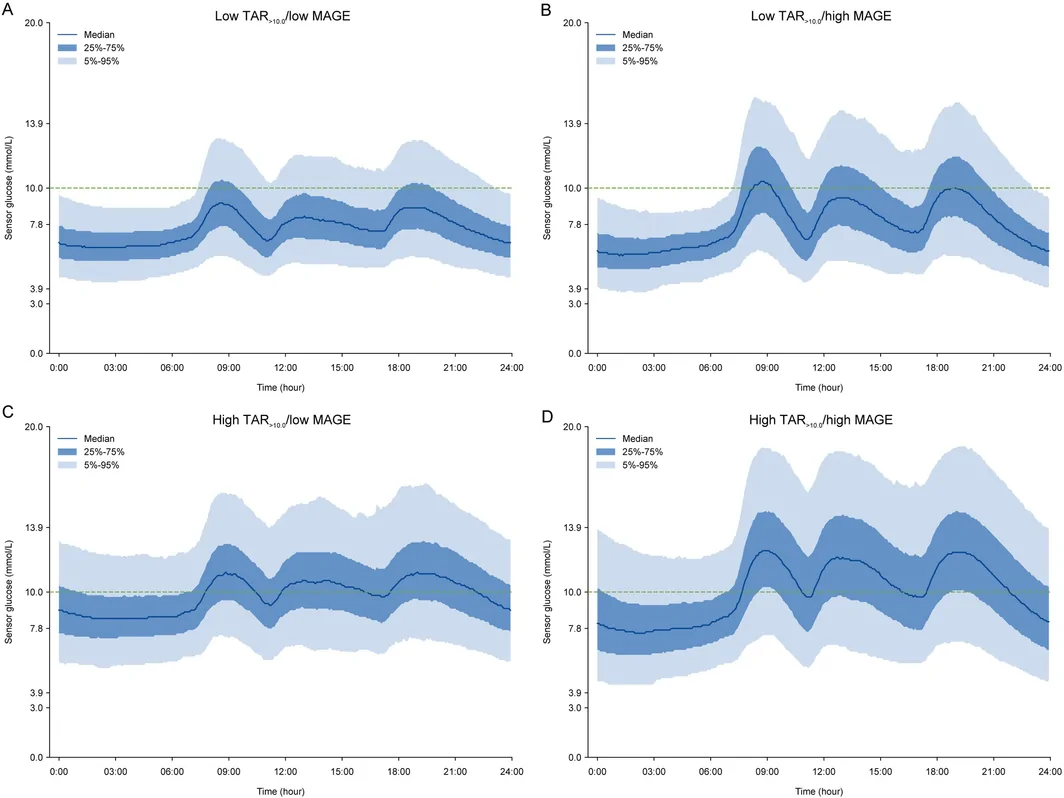
CGM profiles across glycemic phenotypes. The median cutoffs used to define the four glycemic phenotypes were 27.0% for TAR_>10.0_ and 5.3 mmol/L (95 mg/dL) for MAGE. Abbreviations: CGM, continuous glucose monitoring; MAGE, mean amplitude of glycemic excursions; TAR_>10.0_, time above range > 10.0 mmol/L (> 180 mg/dL).

**FIGURE 3:**
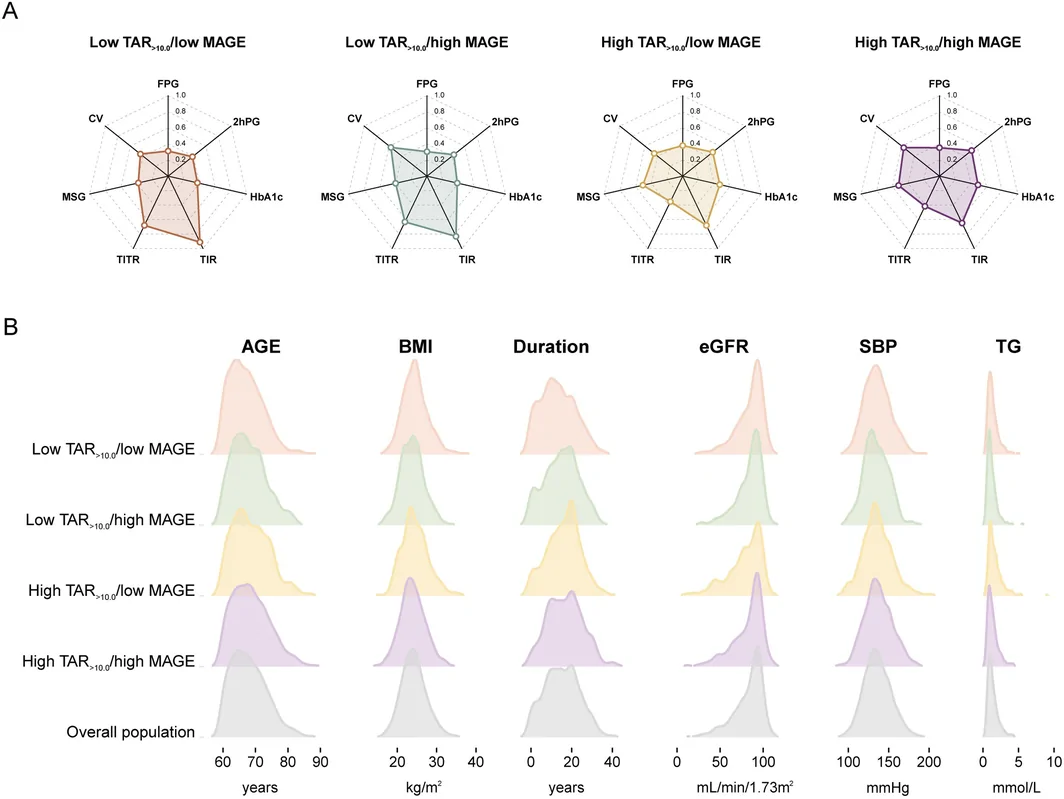
Glycemic and clinical profiles across CGM‐derived glycemic phenotypes. The median cutoffs used to define the four glycemic phenotypes were 27.0% for TAR_>10.0_ and 5.3 mmol/L (95 mg/dL) for MAGE. Abbreviations: 2hPG, 2‐h postprandial plasma glucose; BMI, body mass index; CGM, continuous glucose monitoring; CV, coefficient of variation; eGFR, estimated glomerular filtration rate; FPG, fasting plasma glucose; HbA1c, glycated hemoglobin A1c; MAGE, mean amplitude of glycemic excursions; MSG, mean sensor glucose; SBP, systolic blood pressure; TAR_>10.0_, time above range > 10.0 mmol/L (> 180 mg/dL); TG, triglycerides; TIR, time in range; TITR, time in tight range.

Compared with low TAR_>10.0_ phenotypes, the two high TAR_>10.0_ phenotypes were characterized by older age and longer diabetes duration (both *p* < 0.001), but showed distinct glycemic and metabolic patterns. The high TAR_>10.0_/high MAGE phenotype had the poorest overall glycemic control and the greatest glucose variability, with the highest HbA1c [9.1% (8.0%–10.3%)] and 2hPG [13.4 (10.6–16.3) mmol/L; 241 (191–293) mg/dL], as well as the lowest TIR [56.0% (43.8%–65.0%)] among the four groups (all *p* < 0.001). In contrast, the high TAR_>10.0_/low MAGE phenotype showed a fasting hyperglycemia‐dominant pattern. This group had the highest FPG and triglyceride levels but the lowest TITR and eGFR (all *p* < 0.001).

### 3.4 Association Between Glycemic Phenotypes and Advanced CKM Syndrome

Among the four glycemic phenotypes, the high TAR_>10.0_/low MAGE group had the highest prevalence of advanced CKM syndrome (73.3%, *p* overall < 0.001; Figure 4). Using the low TAR_>10.0_/low MAGE group as the reference, both high TAR_>10.0_ phenotypes were significantly associated with higher odds of advanced CKM syndrome in the unadjusted model (Model 1). These associations remained significant after adjustment for age, sex, diabetes duration, BMI, and current smoking status (Model 2), and further adjustment for HbA1c (Model 3). In model 3, the ORs were 1.62 (95% CI 1.16–2.27) for the high TAR_>10.0_/low MAGE group and 1.35 (95% CI 1.04–1.76) for the high TAR_>10.0_/high MAGE group. After additional adjustment for glucose‐lowering treatment, the association for the high TAR_>10.0_/low MAGE phenotype with advanced CKM syndrome remained significant (OR 1.51, 95% CI 1.07–2.12), although the effect estimate was modestly attenuated (Supplementary Table 4). Similar results were observed for CKM Stage 4, with the high TAR_>10.0_/low MAGE phenotype showing the numerically strongest association among the four phenotypes [OR 1.85 (95% CI 1.30–2.65), *p* < 0.001].

**FIGURE 4:**
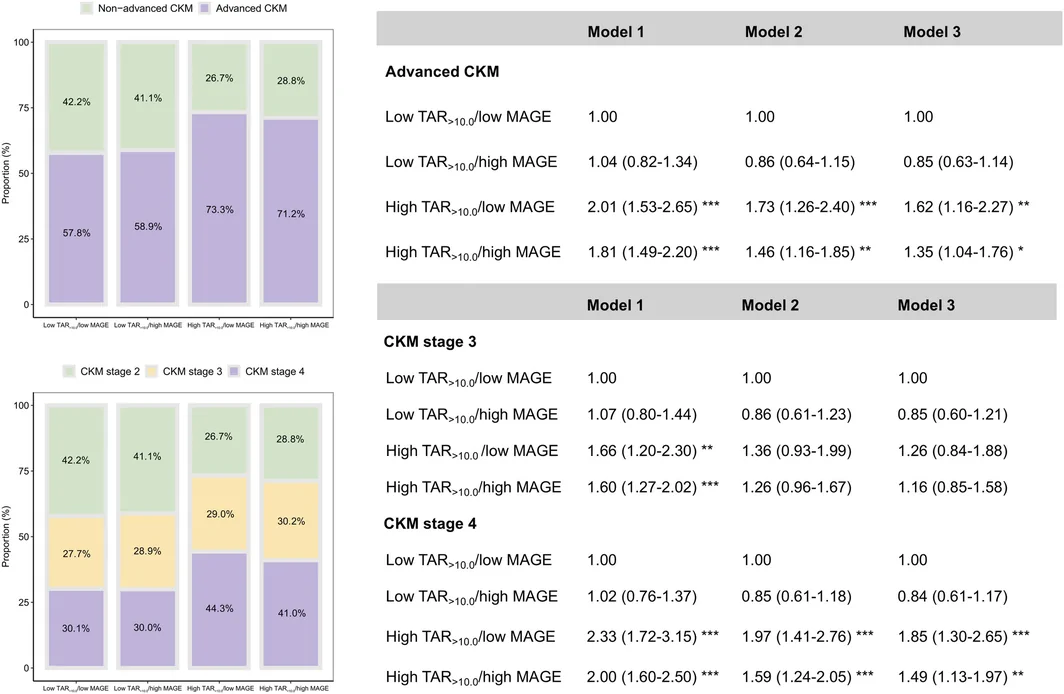
Associations of CGM‐derived glycemic phenotypes with advanced CKM syndrome. Stacked bar plots show the distribution of CKM stages across CGM‐derived glycemic phenotypes. Logistic regression models show the associations of glycemic phenotypes with advanced CKM syndrome, CKM Stage 3, and CKM Stage 4, using the low TAR_>10.0_/low MAGE group as the reference. The median cutoffs used to define the four glycemic phenotypes were 27.0% for TAR_>10.0_ and 5.3 mmol/L (95 mg/dL) for MAGE. Data are presented as ORs and 95% CIs. Model 1 was unadjusted; Model 2 was adjusted for age, sex, diabetes duration, BMI, and current smoking status; Model 3 was further adjusted for HbA1c. Abbreviations: BMI, body mass index; CGM, continuous glucose monitoring; CI, confidence interval; CKM, cardiovascular‐kidney‐metabolic; HbA1c, glycated hemoglobin A1c; MAGE, mean amplitude of glycemic excursions; TAR_>10.0_, time above range > 10.0 mmol/L (> 180 mg/dL). *p < 0.05; **p < 0.01; ***p < 0.001.

Given its close relationship with CKM syndrome, HOMA2‐IR was additionally assessed in participants with valid values. The association between the high TAR_>10.0_/low MAGE phenotype and advanced CKM syndrome was modestly attenuated after further adjustment for HOMA2‐IR, with the OR changing from 1.46 (95% CI 1.02–2.08) to 1.39 (95% CI 0.97–2.00; Supplementary Table 5). In the overall study population, insulin‐related indices differed across the four CGM‐derived phenotypes, with higher HOMA2‐IR and lower HOMA2‐S in the high TAR_>10.0_/low MAGE phenotype (both *p* overall < 0.001; Table 2).

**TABLE 2:** Insulin‐related indices across CGM‐derived glycemic phenotypes.

	Total	Low TAR_>10.0_/low MAGE	Low TAR_>10.0_/high MAGE	High TAR_>10.0_/low MAGE	High TAR_>10.0_/high MAGE	*p* overall
*N*	2491	882	360	345	904	
HbA1c, %	8.1 (7.0, 9.6)	7.2 (6.5, 8.2)	7.4 (6.7, 8.6)	8.8 (7.9, 10.1)	9.1 (8.0, 10.3)	< 0.001
FPG, mmol/L	6.5 (5.4, 7.9)	6.0 (5.3, 7.0)	5.8 (5.0, 6.7)	7.9 (6.5, 9.6)	7.2 (5.8, 8.8)	< 0.001
2hPG, mmol/L	10.9 (8.5, 13.8)	9.0 (7.3, 10.9)	10.4 (8.2, 12.4)	12.2 (9.8, 14.4)	13.4 (10.6, 16.3)	< 0.001
FINS, μU/mL	11.9 (6.5, 20.4)	9.6 (5.6, 16.0)	10.6 (5.6, 17.4)	15.5 (8.6, 26.2)	14.5 (8.4, 23.3)	< 0.001
2hINS, μU/mL	47.5 (28.6, 74.9)	43.9 (25.7, 69.1)	50.0 (33.6, 84.7)	48.0 (25.3, 75.0)	50.6 (32.1, 76.2)	< 0.001
FCP, ng/mL	1.6 (1.0, 2.4)	1.9 (1.3, 2.7)	1.5 (1.0, 2.2)	1.8 (1.2, 2.6)	1.2 (0.8, 1.9)	< 0.001
2hCP, ng/mL	4.0 (2.4, 6.3)	5.2 (3.3, 7.4)	4.4 (2.7, 7.2)	3.6 (2.3, 5.4)	3.0 (2.0, 4.7)	< 0.001
HOMA2‐B	62.7 (41.2, 90.7)	80.8 (58.8, 110.9)	76.3 (54.4, 101.8)	48.3 (33.5, 73.0)	46.7 (31.2, 66.4)	< 0.001
HOMA2‐IR	1.3 (0.8, 1.9)	1.5 (1.0, 2.1)	1.2 (0.7, 1.7)	1.5 (1.0, 2.3)	1.0 (0.6, 1.6)	< 0.001
HOMA2‐S	79.3 (51.8, 127.8)	67.3 (47.3, 101.2)	85.3 (59.0, 134.3)	66.0 (44.0, 99.4)	97.0 (61.2, 158.3)	< 0.001

*Note:* Data are presented as median (interquartile range) or number (percentage) unless otherwise indicated. Values outside the accepted input ranges for HOMA2 calculation were capped at the corresponding upper or lower limits.

Abbreviations: 2hCP, 2‐h C‐peptide; 2hINS, 2‐h insulin; 2hPG, 2‐h postprandial plasma glucose; CGM, continuous glucose monitoring; FCP, fasting C‐peptide; FINS, fasting insulin; FPG, fasting plasma glucose; HbA1c, glycated hemoglobin A1c; HOMA2, homeostasis model assessment 2; MAGE, mean amplitude of glycemic excursions; TAR_>10.0_, time above range > 10.0 mmol/L (> 180 mg/dL).

## 4 Discussion

This study found that CGM‐derived glycemic phenotypes differed significantly in metabolic characteristics and their associations with advanced CKM syndrome. Notably, although the high TAR_>10.0_/low MAGE phenotype did not exhibit the poorest overall glycemic control, it was characterized by greater fasting hyperglycemia and insulin resistance. This phenotype showed the largest point estimate of association with advanced CKM syndrome after adjustment for HbA1c. Clinically interpretable glycemic phenotypes derived from CGM metrics may provide additional information on metabolic heterogeneity and its association with advanced CKM syndrome among older adults with Type 2 diabetes.

Current efforts to characterize phenotypes within the CKM syndrome framework have mainly focused on metabolic features. In studies of obesity phenotypes [22, 23], sarcopenic obesity has been associated with advanced CKM syndrome and subsequent cardiovascular events [23]. Insulin resistance phenotypes, represented by the triglyceride‐glucose (TyG) index and its derived indices, have also been used to identify individuals at higher risk of cardiovascular events and mortality [24, 25, 26, 27]. In addition, a data‐driven cluster analysis identified distinct CKM syndrome clusters, including liver high‐risk, cerebrovascular high‐risk, age‐driven high‐risk, and cardiorenal high‐risk clusters, with heterogeneous organ‐specific outcome profiles [28]. By contrast, evidence regarding glycemic phenotypes within the CKM framework remains lacking.

The concept of glycemic phenotypes emphasizes interindividual pathophysiological heterogeneity reflected in overall patterns of glucose excursions [29, 30]. The advent of CGM has further expanded research on glycemic phenotypes. Hall et al. [3] first used CGM to classify individuals into distinct patterns characterized by low, moderate, or severe glycemic variability, and observed that some individuals classified as normoglycemic by conventional measures still exhibited marked glycemic excursions and transient hyperglycemic exposure. Another study identified three CGM‐derived glycemic phenotypes, which were associated with diabetic kidney disease and diabetic retinopathy [31]. Our previous work also used cluster analysis in individuals with Type 2 diabetes and identified distinct phenotypes that differed in HbA1c, pancreatic β‐cell function, and insulin sensitivity [5]. In Type 1 diabetes, different glycemic phenotypes have likewise been shown to be related to metabolic status [6, 7, 32]. Metwally et al. [8] further used machine learning analysis to identify distinct metabolic subphenotypes in individuals with early dysglycemia, including muscle insulin resistance, hepatic insulin resistance, β‐cell dysfunction, and impaired incretin action, suggesting that CGM‐derived information may reflect metabolic heterogeneity. More recent work based on self‐supervised pretraining using CGM data has also explored the potential use of dynamic CGM information for metabolic profiling and risk stratification [33].

To our knowledge, this is the first study to evaluate glycemic phenotypes within the CKM syndrome framework. Unlike previous studies focusing on individual diabetes‐related complications, the CKM framework captures the coexistence of metabolic, kidney, and cardiovascular abnormalities within a multisystem disease continuum. This framework provides a broader context for evaluating CGM‐derived glycemic phenotypes in relation to cardiometabolic burden beyond individual complication assessments. In the present study, the high TAR_>10.0_/low MAGE phenotype was associated with advanced CKM syndrome and was characterized by an adverse insulin resistance‐related profile. After further adjustment for HOMA2‐IR, its association with advanced CKM syndrome was attenuated, whereas the association for the high TAR_>10.0_/high MAGE phenotype became stronger. These findings suggest that insulin resistance may be related to the association between the high TAR_>10.0_/low MAGE phenotype and advanced CKM syndrome. In line with our findings, Wu et al. [34] reported that patients with Type 2 diabetes and a higher TyG index showed sustained hyperglycemia with relatively low glycemic variability on CGM. One possible explanation is that sustained or fasting‐dominant hyperglycemia may be consistent with impaired insulin‐mediated suppression of hepatic glucose production and reduced peripheral glucose uptake [35], resulting in prolonged hyperglycemic exposure with relatively modest variability on CGM. Given its links to atherosclerosis, dyslipidemia, and renal hemodynamic abnormalities [36], insulin resistance may represent a shared pathophysiological feature associated with both this glycemic phenotype and advanced CKM syndrome. CGM‐derived phenotypes may provide additional information on insulin resistance‐related metabolic heterogeneity beyond isolated glycemic indicators [37, 38, 39]. Future studies should assess whether higher‐dimensional CGM analyses can support diabetes subtype differentiation and complication prediction.

Our study has several strengths, including a relatively large sample size, comprehensive data on CGM metrics, conventional glycemic indices including fasting and postprandial glucose, and variables required for PREVENT risk score calculation and chronic kidney disease assessment. Data collection followed standardized procedures. Several limitations should also be acknowledged. First, the cross‐sectional design limits the assessment of temporal and causal relationships between glycemic phenotypes and advanced CKM syndrome. Repeated kidney function and albuminuria measurements were not systematically available, limiting confirmation of CKD chronicity. Prospective longitudinal studies incorporating repeated CGM assessments and follow‐up of CKM progression and cardiorenal outcomes are needed to validate these findings. Second, the median CGM wear duration was 7 days, which was shorter than the 14 days recommended by guidelines but can still provide reasonably reliable glycemic assessment [40, 41]. Longer or repeated CGM assessments may improve the stability of variability‐related metrics and phenotype classification. Third, the study population consisted of hospitalized patients with Type 2 diabetes. Although admissions were primarily for standardized clinical evaluation and patients with malignancy, severe hepatic dysfunction, or dialysis dependence were excluded, the inpatient setting may have included a higher proportion of individuals with a greater burden of diabetes‐related conditions and introduced selection bias. Therefore, the generalizability of our findings to community‐based and outpatient settings requires further validation. Fourth, the outcome‐guided classification and its association with advanced CKM syndrome were evaluated within the same study population. Therefore, selection‐induced optimism cannot be excluded, and independent validation is warranted.

In conclusion, this study constructed simplified and clinically interpretable CGM‐derived glycemic phenotypes in older adults with Type 2 diabetes. The four phenotypes demonstrated distinct metabolic profiles and differential associations with advanced CKM syndrome. These findings suggest that CGM‐derived glycemic patterns may provide additional information for characterizing metabolic heterogeneity among older adults with Type 2 diabetes.

## Author Contributions

F.J. and J.Z. conceived and designed the study, contributed to the interpretation of the data and revision of the manuscript, have full access to all the data in the study, and take responsibility for the integrity of the data and the accuracy of the data analysis. H.S. and J.N. performed statistical analysis and wrote the draft of the manuscript. J.C. cleaned the data and contributed to manuscript preparation. J.G. contributed to the statistical methodology and analysis. X.M. and Y.W. contributed to data collection. J.L. reviewed and edited the manuscript. All authors read and approved the final manuscript.

## Funding

This work was funded by Noncommunicable Chronic Diseases‐National Science and Technology Major Project (2024ZD0532000, 2024ZD0532001), Shanghai Leading Talent Program of Eastern Talent Plan (LJ2024121), Shenzhen Medical Research Fund (C2406002), and Sanming Project of Medicine in Shenzhen (SZSM202311019).

## Ethics Statement

This study was approved by the Ethics Committee of Shanghai Sixth People's Hospital Affiliated to Shanghai Jiao Tong University School of Medicine and was in accordance with the 1964 Declaration of Helsinki and its later amendments or comparable ethical standards (approval No. 2020‐KY‐024[K]). Informed consent was obtained from all individual participants included in the study.

## Conflicts of Interest

The authors declare no conflicts of interest.

## Supporting information


**Supplementary Table 1.** Definitions and calculation methods of CGM‐derived metrics.
**Supplementary Table 2.** Definitions of CKM syndrome stages.
**Supplementary Table 3.** Clinical characteristics of participants across CGM‐derived glycemic phenotypes.
**Supplementary Table 4.** Association between CGM‐derived glycemic phenotypes and advanced CKM syndrome after adjustment for glucose‐lowering treatment.
**Supplementary Table 5.** Associations of CGM‐derived glycemic phenotypes with advanced CKM syndrome in participants with valid HOMA2‐IR values (*N* = 2240).

## Data Availability

Restrictions apply to the availability of data generated or analyzed during this study to preserve patient confidentiality or because they were used under license. Data are however available from the authors upon reasonable request.
