# Supplementary material for: Continuous Glucose Monitoring‐Derived Glycemic Phenotypes Reflect Metabolic Heterogeneity Linked to Advanced Cardiovascular‐Kidney‐Metabolic Syndrome in Older Adults With Type 2 Diabetes
Source: J Diabetes. 2026 Nov 10;18(11):e70298. doi: 10.1111/1753-0407.70298 (PMC13751066; doi:10.1111/1753-0407.70298)
Supplement: Supplementary file 1 [file JDB-18-e70298-s001.docx]

**Supplementary Appendix**

[Supplementary Table 1. Definitions and calculation methods of CGM-derived metrics	2](#_Toc210397693)

[Supplementary Table 2. Definitions of CKM syndrome stages	4](#_Toc210397693)

[Supplementary Table 3. Clinical characteristics of participants across CGM-derived glycemic phenotypes	5](#_Toc210397695)

[Supplementary Table 4. Association between CGM-derived glycemic phenotypes and advanced CKM syndrome after adjustment for glucose-lowering treatment	8](#_Toc210397695)

[Supplementary Table 5. Associations of CGM-derived glycemic phenotypes with advanced CKM syndrome in participants with valid HOMA2-IR values (N=2240)	9](#_Toc210397693)

**Supplementary Table 1. Definitions and calculation methods of CGM-derived metrics**

| **Metric** | **Full name** | **Clinical interpretation** | **Definition and calculation** |
| --- | --- | --- | --- |
| TIR | Time in range | Overall glycemic control and time spent within the target glucose range | Percentage of valid CGM readings within 3.9–10.0 mmol/L (70–180 mg/dL). Calculated as the number of readings between 3.9 and 10.0 mmol/L divided by the total number of valid readings × 100%. |
| TITR | Time in tight range | Achievement of a narrower glycemic target | Percentage of valid CGM readings within 3.9–7.8 mmol/L (70–140 mg/dL). Calculated as the number of readings between 3.9 and 7.8 mmol/L divided by the total number of valid readings × 100%. |
| TAR_>7.8_ | Time above range >7.8 mmol/L | Mild hyperglycemic exposure | Percentage of valid CGM readings above 7.8 mmol/L. Calculated as readings >7.8 mmol/L (140 mg/dL) divided by total valid readings × 100%. |
| TAR_>10.0_ | Time above range >10.0 mmol/L | Clinically relevant hyperglycemic burden | Percentage of valid CGM readings above 10.0 mmol/L. Calculated as readings >10.0 mmol/L (180 mg/dL) divided by total valid readings × 100%. |
| TAR_>13.9_ | Time above range >13.9 mmol/L | More severe hyperglycemic exposure | Percentage of valid CGM readings above 13.9 mmol/L. Calculated as readings >13.9 mmol/L (250 mg/dL) divided by total valid readings × 100%. |
| TBR_<3.9_ | Time below range <3.9 mmol/L | Level 1 hypoglycemic exposure | Percentage of valid CGM readings below 3.9 mmol/L. Calculated as readings <3.9 mmol/L (70 mg/dL) divided by total valid readings × 100%. |
| TBR_<3.0_ | Time below range <3.0 mmol/L | Clinically significant hypoglycemic exposure | Percentage of valid CGM readings below 3.0 mmol/L. Calculated as readings <3.0 mmol/L (54 mg/dL) divided by total valid readings × 100%. |
| MSG | Mean sensor glucose | Average glucose exposure | Arithmetic mean of all valid CGM glucose readings during the monitoring period. |
| SD | Standard deviation of sensor glucose | Absolute glycemic dispersion | Standard deviation of all valid CGM glucose readings, reflecting the dispersion of glucose values. |
| CV | Coefficient of variation | Relative glycemic variability independent of mean glucose level | Relative glycemic variability, calculated as SD divided by MSG × 100%. |
| MAGE | Mean amplitude of glycemic excursions | Major intraday glucose excursions and glycemic variability | Mean amplitude of valid glycemic excursions. It is calculated as the mean absolute difference between consecutive glucose peaks and nadirs when the excursion exceeds 1 SD of the mean glucose level. MAGE = Σ\|G_peak,i − G_nadir,i\|/n. |
| IQR | Interquartile range | Distributional spread of glucose values | Difference between the 75th and 25th percentiles of CGM glucose values. |
| LAGE | Largest amplitude of glycemic excursions | Largest observed glucose fluctuation | Difference between the maximum and minimum CGM glucose values during the monitoring period. |
| MAG | Mean absolute glucose change | Average rate of glucose change over time | Mean rate of absolute glucose change over time. Calculated as the sum of absolute differences between consecutive glucose readings divided by the total monitoring time, usually expressed as mmol/L per hour. MAG = Σ\|G(i+1)−G(i)\| / total monitoring time. |
| MODD | Mean of daily differences | Day-to-day glycemic variability | Mean absolute difference between glucose values measured at the same time on two consecutive days, reflecting between-day glycemic variability. |
| CONGA1h | Continuous overall net glycemic action over 1 hour | Short-term glycemic variability over a 1-hour interval | Standard deviation of the differences between each glucose reading and the reading obtained 1 hour earlier. CONGA1h = SD(Gt−Gt−1h). |
| CONGA4h | Continuous overall net glycemic action over 4 hours | Glycemic variability over a 4-hour interval | Standard deviation of the differences between each glucose reading and the reading obtained 4 hours earlier. CONGA4h = SD(Gt−Gt−4h). |
| ADRR | Average daily risk range | Combined risk from hyperglycemia and hypoglycemia | A risk-based metric summarizing both hyperglycemic and hypoglycemic risk. It is calculated from transformed glucose values as the average across days of the sum of the daily maximum high-glucose risk and daily maximum low-glucose risk. $\text{ADRR = 1/D ∑\_(d=1)\textasciicircum D (LR\_d + HR\_d)}$. |
| J-index | J-index | Combined glycemic exposure and variability | Composite index reflecting both mean glucose and glycemic variability. Usually calculated using glucose values in mg/dL as: J-index = 0.001 × (MSG + SD)². |

Notes: The calculation methods of CGM-derived metrics were based on previously established approaches and consensus recommendations (*Service FJ et al., Diabetes. 1970;19:644–655; Rodbard D. Diabetes Technol Ther. 2009;11:S55–S67; Battelino T et al. Lancet Diabetes Endocrinol. 2023;11:42–57*).

**Supplementary Table 2. Definitions of CKM syndrome stages**

| **CKM Syndrome Stages** | **Definition** |
| --- | --- |
| Stage 2 | Individuals with metabolic risk factors:  Diabetes,  Hypertension ^a^,  Elevated fasting serum triglycerides (≥1.5 mmol/L),  Metabolic Syndrome ^b^,  or chronic kidney disease ^c^ (moderate- or high-risk) |
| Stage 3 | Risk equivalents of subclinical CVD:  Very high-risk chronic kidney disease ^c^  High predicted 10-y cardiovascular disease risk (≥20%) ^d^ |
| Stage 4 | Clinical cardiovascular disease (coronary heart disease, myocardial infarction, stroke, or peripheral artery disease) |

^a^ Hypertension is defined as systolic blood pressure ≥130 mmHg or diastolic blood pressure ≥ 80 mmHg or under anti-hypertensive medication.

^b^ Metabolic Syndrome is defined by the presence of three or more of the following: (1) waist circumference ≥80 cm in women or ≥90 cm in men; (2) high-density lipoprotein (HDL) cholesterol <1.03 mmol/L in men or <1.29 mmol/L in women; (3) triglyceride levels ≥1.70 mmol/L; (4) elevated blood pressure (systolic blood pressure ≥130 mmHg or diastolic blood pressure ≥80 mmHg and/or use of antihypertensive medications); and (5) prediabetes.

^c^ Chronic kidney disease (CKD) classification is defined according to the Kidney Disease Improving Global Outcomes (KDIGO) 2012 recommendations. GFR is classified into 5 stages (G1 to G5), while UACR was classified into 3 categories (A1 to A3). Participants with G1-2 and A1 were defined at low risk for CKD; Participants with G1-2 and A2, or G3a and A1 were defined at moderate risk for CKD; Participants with G1-2 and A3, or G3a and A2, or G3b and A1 were defined at high risk for CKD; Participants with G4-5 and A1, or G3b-5 and A2, or G3a-5 and A3 were defined at very high risk for CKD.

^d^ Predicted 10-year cardiovascular disease (CVD) risk is calculated using the PREVENT equations (AHA Predicting Risk of CVD Events). The PREVENT model is developed and validated for adults aged 30–79 years; individuals aged ≥80 years are assigned an age of 79 years to provide conservative estimates of 10-year CVD risk. The PREVENT equations are calibrated for the following variable ranges: total cholesterol 3.37–8.29 mmol/L, HDL cholesterol 0.52–2.59 mmol/L, systolic blood pressure 90–200 mmHg, and GFR 14–140 mL/min/1.73 m². Values outside these ranges are truncated to the corresponding upper or lower limits to approximate PREVENT risk categories.

**Supplementary Table 3. Clinical characteristics of participants across CGM-derived glycemic phenotypes**

|  | **Low TAR_>10.0_/low MAGE** | **Low TAR_>10.0_/high MAGE** | **High TAR_>10.0_/low MAGE** | **High TAR_>10.0_/high MAGE** | ***P* overall** |
| --- | --- | --- | --- | --- | --- |
| N | 882 | 360 | 345 | 904 |  |
| Sex, *n* (%) |  |  |  |  | 0.338 |
| Male | 453 (51.4%) | 200 (55.6%) | 179 (51.9%) | 497 (55.0%) |  |
| Female | 429 (48.6%) | 160 (44.4%) | 166 (48.1%) | 407 (45.0%) |  |
| Age, years | 67.0 (63.0, 70.0) | 67.0 (64.0, 71.0) | 68.0 (64.0, 72.0) | 68.0 (64.0, 72.0) | <0.001 |
| Diabetes duration, years | 13.0 (7.0, 20.0) | 15.0 (9.0, 20.0) | 17.0 (10.0, 21.0) | 17.0 (10.0, 23.0) | <0.001 |
| Systolic blood pressure, mmHg | 135.0 (124.0, 146.0) | 133.5 (124.0, 145.0) | 135.0 (124.0, 148.0) | 136.0 (126.0, 150.0) | 0.064 |
| Diastolic blood pressure, mmHg | 80.0 (73.0, 85.0) | 78.0 (72.0, 84.0) | 80.0 (72.0, 85.0) | 78.0 (70.8, 84.0) | 0.006 |
| Body mass index, kg/m^2^ | 24.4 (22.3, 26.3) | 23.7 (21.7, 25.7) | 24.2 (22.3, 26.6) | 23.7 (21.9, 26.0) | <0.001 |
| eGFR, mL/min/1.73 m^2^ | 89.0 (76.2, 95.1) | 87.6 (76.8, 94.4) | 83.1 (67.9, 93.6) | 88.4 (72.8, 94.3) | <0.001 |
| Total cholesterol, mmol/L | 4.2 (3.5, 5.0) | 4.4 (3.7, 5.1) | 4.5 (3.6, 5.2) | 4.4 (3.6, 5.3) | 0.007 |
| Triglycerides, mmol/L | 1.2 (0.9, 1.7) | 1.1 (0.8, 1.5) | 1.4 (1.0, 2.1) | 1.2 (0.8, 1.7) | <0.001 |
| HDL cholesterol, mmol/L | 1.1 (0.9, 1.3) | 1.2 (1.0, 1.4) | 1.0 (0.9, 1.2) | 1.1 (0.9, 1.4) | <0.001 |
| LDL cholesterol, mmol/L | 2.3 (1.8, 3.1) | 2.5 (1.8, 3.0) | 2.5 (1.9, 3.1) | 2.4 (1.9, 3.1) | 0.068 |
| HbA1c, % | 7.2 (6.5, 8.2) | 7.4 (6.7, 8.6) | 8.8 (7.9, 10.1) | 9.1 (8.0, 10.3) | <0.001 |
| FPG, mmol/L | 6.0 (5.3, 7.0) | 5.8 (5.0, 6.7) | 7.9 (6.5, 9.6) | 7.2 (5.8, 8.8) | <0.001 |
| 2hPG, mmol/L | 9.0 (7.3, 10.9) | 10.4 (8.2, 12.4) | 12.2 (9.8, 14.4) | 13.4 (10.6, 16.3) | <0.001 |
| Current smokers, *n* (%) | 126 (14.3%) | 64 (17.8%) | 53 (15.4%) | 171 (18.9%) | 0.054 |
| Oral glucose-lowering drugs, *n* (%) | 673 (76.3%) | 222 (61.7%) | 200 (58.0%) | 359 (39.7%) | <0.001 |
| Metformin, *n* (%) | 449 (50.9%) | 136 (37.8%) | 101 (29.3%) | 187 (20.7%) | <0.001 |
| Sulfonylureas, *n* (%) | 103 (11.7%) | 62 (17.2%) | 23 (6.7%) | 36 (4.0%) | <0.001 |
| Thiazolidinediones, *n* (%) | 21 (2.4%) | 9 (2.5%) | 3 (0.9%) | 16 (1.8%) | 0.302 |
| Glinides, *n* (%) | 29 (3.3%) | 15 (4.2%) | 19 (5.5%) | 19 (2.1%) | 0.017 |
| DPP-4 inhibitors, *n* (%) | 205 (23.2%) | 51 (14.2%) | 51 (14.8%) | 60 (6.6%) | <0.001 |
| α-Glucosidase inhibitors, *n* (%) | 340 (38.5%) | 87 (24.2%) | 114 (33.0%) | 158 (17.5%) | <0.001 |
| SGLT2 inhibitors, *n* (%) | 209 (23.7%) | 69 (19.2%) | 53 (15.4%) | 82 (9.1%) | <0.001 |
| Insulin, *n* (%) | 421 (47.7%) | 233 (64.7%) | 291 (84.3%) | 830 (91.8%) | <0.001 |
| GLP-1 receptor agonist, *n* (%) | 38 (4.3%) | 7 (1.9%) | 18 (5.2%) | 9 (1.0%) | <0.001 |
| Antihypertensive agents, *n* (%) | 473 (53.6%) | 182 (50.6%) | 211 (61.2%) | 506 (56.0%) | 0.027 |
| Lipid‐lowering agents, *n* (%) | 652 (73.9%) | 268 (74.4%) | 271 (78.6%) | 687 (76.0%) | 0.357 |
| Antiplatelet agents, *n* (%) | 272 (30.8%) | 110 (30.6%) | 133 (38.6%) | 339 (37.5%) | 0.003 |
| Mean sensor glucose, mmol/L | 7.7 (7.0, 8.2) | 8.0 (7.5, 8.4) | 9.7 (9.3, 10.4) | 9.9 (9.3, 11.0) | <0.001 |
| Standard deviation, mmol/L | 1.7 (1.4, 2.0) | 2.5 (2.2, 2.8) | 2.3 (2.0, 2.7) | 3.1 (2.8, 3.6) | <0.001 |
| Coefficient of variation, % | 22.7 (19.6, 26.4) | 31.3 (28.0, 35.3) | 23.6 (20.4, 26.9) | 30.9 (27.4, 35.4) | <0.001 |
| MAGE, mmol/L | 4.0 (3.3, 4.6) | 6.2 (5.7, 6.9) | 4.5 (4.0, 4.9) | 6.8 (6.1, 8.2) | <0.001 |
| TAR_>13.9_, % | 0.0 (0.0, 1.0) | 2.0 (1.0, 5.0) | 5.0 (2.0, 10.0) | 13.0 (7.0, 20.0) | <0.001 |
| TAR_>10.0_, % | 10.0 (5.0, 16.0) | 20.0 (15.0, 23.0) | 40.0 (33.0, 52.0) | 43.0 (34.0, 56.0) | <0.001 |
| TAR_>7.8_, % | 40.0 (26.0, 51.8) | 44.0 (36.8, 50.0) | 80.0 (72.0, 88.0) | 72.0 (61.0, 83.0) | <0.001 |
| TIR, % | 89.0 (83.0, 94.0) | 79.0 (75.0, 83.0) | 60.0 (48.0, 67.0) | 56.0 (43.8, 65.0) | <0.001 |
| TITR, % | 60.0 (48.0, 73.0) | 54.0 (48.0, 62.0) | 19.0 (12.0, 28.0) | 27.0 (16.0, 38.0) | <0.001 |
| TBR_<3.9_, % | 0.0 (0.0, 1.0) | 1.0 (0.0, 3.0) | 0.0 (0.0, 0.0) | 0.0 (0.0, 1.0) | <0.001 |
| TBR_<3.0_, % | 0.0 (0.0, 0.0) | 0.0 (0.0, 0.0) | 0.0 (0.0, 0.0) | 0.0 (0.0, 0.0) | <0.001 |

Notes: Data are presented as median (interquartile range) or number (percentage) unless otherwise indicated. The median cutoffs used to define the four glycemic phenotypes were 27.0% for TAR_>10.0_ and 5.3 mmol/L (95 mg/dL) for MAGE.

Abbreviations: 2hPG, 2-hour plasma glucose; eGFR, estimated glomerular filtration rate; FPG, fasting plasma glucose; HDL, high-density lipoprotein; GLP-1, glucagon-like peptide-1; HbA1c, glycated hemoglobin A1c; LDL, low-density lipoprotein; MAGE, mean amplitude of glycemic excursions; SD, standard deviation; TAR_>13.9_, time above range >13.9 mmol/L (>250 mg/dL); TAR_>10.0_, time above range >10.0 mmol/L (>180 mg/dL); TAR_>7.8_, time above range >7.8 mmol/L (>140 mg/dL); TBR_<3.9_, time below range <3.9 mmol/L (<70 mg/dL); TBR_<3.0_, time below range <3.0 mmol/L (<54 mg/dL); TITR, time in tight range; TIR, time in range.

**Supplementary Table 4. Association between CGM-derived glycemic phenotypes and advanced CKM syndrome after adjustment for glucose-lowering treatment**

|  | **Adjusted OR (95% CI)** |
| --- | --- |
| **Advanced CKM** |  |
| Low TAR_>10.0_/low MAGE | 1.00 |
| Low TAR_>10.0_/high MAGE | 0.82 (0.61-1.11) |
| High TAR_>10.0_/low MAGE | 1.51 (1.07-2.12) ^*^ |
| High TAR_>10.0_/high MAGE | 1.25 (0.95-1.64) |
| **CKM stage 3** |  |
| Low TAR_>10.0_/low MAGE | 1.00 |
| Low TAR_>10.0_/high MAGE | 0.84 (0.59-1.20) |
| High TAR_>10.0_/low MAGE | 1.20 (0.80-1.79) |
| High TAR_>10.0_/high MAGE | 1.09 (0.79-1.51) |
| **CKM stage 4** |  |
| Low TAR_>10.0_/low MAGE | 1.00 |
| Low TAR_>10.0_/high MAGE | 0.81 (0.58-1.13) |
| High TAR_>10.0_/low MAGE | 1.70 (1.19-2.44) ^**^ |
| High TAR_>10.0_/high MAGE | 1.35 (1.01-1.81) ^*^ |

Notes: Data are presented as ORs and 95% CIs. The model was adjusted for age, sex, diabetes duration, BMI, current smoking status, HbA1c, insulin use, and oral glucose-lowering drug use. The low TAR_>10.0_/low MAGE phenotype was used as the reference group. ^*^*P* < 0.05, ^**^*P* < 0.01.

Abbreviations: BMI, body mass index; CGM, continuous glucose monitoring; CI, confidence interval; CKM, cardiovascular-kidney-metabolic; HbA1c, glycated hemoglobin A1c; MAGE, mean amplitude of glycemic excursions; OR, odds ratio; TAR_>10.0_, time above range >10.0 mmol/L (>180 mg/dL).

**Supplementary Table 5. Associations of CGM-derived glycemic phenotypes with advanced CKM syndrome in participants with valid HOMA2-IR values (N=2240)**

|  | **Model 1** | **Model 2** | **Model 3** | **Model 4** |
| --- | --- | --- | --- | --- |
| **Advanced CKM** |  |  |  |  |
| Low TAR_>10.0_/low MAGE | 1.00 | 1.00 | 1.00 | 1.00 |
| Low TAR_>10.0_/high MAGE | 1.02 (0.78-1.34) | 0.84 (0.61-1.16) | 0.83 (0.60-1.14) | 0.90 (0.65-1.24) |
| High TAR_>10.0_/low MAGE | 1.93 (1.46-2.57) ^***^ | 1.62 (1.16-2.27) ^**^ | 1.46 (1.02-2.08) ^*^ | 1.39 (0.97-2.00) |
| High TAR_>10.0_/high MAGE | 1.86 (1.51-2.29) ^***^ | 1.50 (1.17-1.92) ^**^ | 1.34 (1.02-1.76) ^*^ | 1.43 (1.09-1.89) ^*^ |
| **CKM stage 3** |  |  |  |  |
| Low TAR_>10.0_/low MAGE | 1.00 | 1.00 | 1.00 | 1.00 |
| Low TAR_>10.0_/high MAGE | 1.01 (0.73-1.40) | 0.82 (0.56-1.21) | 0.81 (0.55-1.19) | 0.86 (0.58-1.27) |
| High TAR_>10.0_/low MAGE | 1.80 (1.29-2.51) ^***^ | 1.46 (0.98-2.16) | 1.31 (0.87-1.98) | 1.27 (0.83-1.93) |
| High TAR_>10.0_/high MAGE | 1.72 (1.35-2.20) ^***^ | 1.37 (1.02-1.83) ^*^ | 1.22 (0.88-1.68) | 1.29 (0.94-1.79) |
| **CKM stage 4** |  |  |  |  |
| Low TAR_>10.0_/low MAGE | 1.00 | 1.00 | 1.00 | 1.00 |
| Low TAR_>10.0_/high MAGE | 1.03 (0.75-1.41) | 0.85 (0.60-1.21) | 0.84 (0.59-1.19) | 0.92 (0.64-1.31) |
| High TAR_>10.0_/low MAGE | 2.05 (1.49-2.82) ^***^ | 1.71 (1.20-2.45) ^**^ | 1.55 (1.06-2.26) ^*^ | 1.47 (1.00-2.15) ^*^ |
| High TAR_>10.0_/high MAGE | 1.98 (1.57-2.51) ^***^ | 1.59 (1.22-2.07) ^***^ | 1.42 (1.06-1.90) ^*^ | 1.53 (1.14-2.06) ^**^ |

Notes: Model 1 was unadjusted; Model 2 was adjusted for age, sex, diabetes duration, BMI, and current smoking; Model 3 was further adjusted for HbA1c; Model 4 further adjusted for HOMA2-IR. Analyses were restricted to participants within the valid input ranges of the HOMA2 calculator: FPG, 3.0–25.0 mmol/L (54–450 mg/dL); FCP, 0.2–3.5 nmol/L. ^*^*P* < 0.05; ^**^*P* < 0.01; ^***^*P* < 0.001.

Abbreviations: BMI, body mass index; CGM, continuous glucose monitoring; CKM, cardiovascular-kidney-metabolic; FCP, fasting C-peptide; FPG, fasting plasma glucose; HbA1c, glycated hemoglobin A1c; HOMA2, homeostasis model assessment 2; MAGE, mean amplitude of glycemic excursions; TAR_>10.0_, time above range >10.0 mmol/L (>180 mg/dL).
